# CellTag Indexing: genetic barcode-based sample multiplexing for single-cell genomics

**DOI:** 10.1186/s13059-019-1699-y

**Published:** 2019-05-09

**Authors:** Chuner Guo, Wenjun Kong, Kenji Kamimoto, Guillermo C. Rivera-Gonzalez, Xue Yang, Yuhei Kirita, Samantha A. Morris

**Affiliations:** 1 Department of Developmental Biology, Washington University School of Medicine in St Louis, 660 S. Euclid Avenue, Campus Box 8103, St. Louis, MO 63110 USA; 2 Department of Genetics, Washington University School of Medicine in St. Louis, 660 S. Euclid Avenue, Campus Box 8103, St. Louis, MO 63110 USA; 3 Center of Regenerative Medicine, Washington University School of Medicine in St. Louis, 660 S. Euclid Avenue, Campus Box 8103, St. Louis, MO 63110 USA; 4 Division of Nephrology, Department of Medicine, Washington University School of Medicine in St. Louis, 660 S. Euclid Avenue, Campus Box 8103, St. Louis, MO 63110 USA

## Abstract

High-throughput single-cell assays increasingly require special consideration in experimental design, sample multiplexing, batch effect removal, and data interpretation. Here, we describe a lentiviral barcode-based multiplexing approach, CellTag Indexing, which uses predefined genetic barcodes that are heritable, enabling cell populations to be tagged, pooled, and tracked over time in the same experimental replicate. We demonstrate the utility of CellTag Indexing by sequencing transcriptomes using a variety of cell types, including long-term tracking of cell engraftment and differentiation in vivo. Together, this presents CellTag Indexing as a broadly applicable genetic multiplexing tool that is complementary with existing single-cell technologies.

**Electronic supplementary material:**

The online version of this article (10.1186/s13059-019-1699-y) contains supplementary material, which is available to authorized users.

## Introduction

Single-cell technology is advancing at a rapid pace, providing unique opportunities to investigate biological systems and processes with unparalleled resolution. As an increasing variety of assays are being deployed at single-cell resolution, this has presented new challenges for experimental design and data analysis. Recently, batch effects were shown to drive aberrant clustering of the same biological sample processed via two different methodologies [1], demonstrating how the accuracy of single-cell data analysis can be confounded by measurement errors. Several algorithms currently exist to support the computational correction of batch effects [2–5]. These methods aim to minimize technical artifacts by regressing out known factors of variation during single-cell data processing. However, this requires prior knowledge of the specific factors contributing to batch effects, limiting these approaches. In an alternative strategy, samples are pooled together and subsequently demultiplexed, based on their natural genetic variation [6], a powerful approach that supports the multiplexing of up to ~ 20 samples. However, if the samples are not genetically distinct or are not accompanied by detailed genotypic knowledge, demultiplexing by genetic variation does not represent a feasible approach. For instance, this strategy would not be suitable for comparing different experimental groups from the same individual or animal model where genetic background stays constant.

Recently, several “label-and-pool” approaches have been developed to mark individual cells of the same sample with a distinct barcode prior to pooling and processing in the same single-cell RNA-sequencing (scRNA-seq) run [7–12]. For example, cells can be tagged with barcoded antibodies [9, 12], chemically labeled with DNA oligonucleotides [8, 10], or transiently transfected with DNA oligonucleotides [11], such that sample identifiers for each cell can be read, in parallel with their transcriptomes. Similarly, several other methods exist to couple genetic perturbations with barcodes [13–17], although these have not been demonstrated to support reliable, large-scale sample multiplexing. Here, we introduce a methodology to multiplex biological samples via long-term genetic labeling with heritable virally delivered barcodes, “CellTags.” In this approach, defined 8-nucleotide (nt) CellTag barcodes are expressed as polyadenylated transcripts, captured in standard single-cell processing protocols. This design permits the indelible labeling and subsequent identification of cells by sample, in parallel with the measurement of their identity and state. In contrast to labeling approaches based on transient physical interactions at the cell or nuclear surface, CellTag Indexed cells retain their heritable barcodes for an extended period in vitro and in vivo, supporting long-term cell tracking experiments. This also distinguishes CellTag Indexing as a unique multiplexing tool in that cell samples can be tagged, mixed and tracked within the same biological replicate, and processed together to mitigate unwanted biological and technical variation.

Here, we validate CellTag Index-based multiplexing via the labeling and mixing of genetically distinct populations, demonstrating accurate and efficient demultiplexing of sample identity. Furthermore, we demonstrate the efficacy of CellTag Indexing for long-term live cell multiplexing, via the establishment of a unique competitive transplant model. In this context, we showcase how CellTag Indexing can be used for in vivo multiplexing to precisely quantify engraftment and differentiation potential of distinct, competing cell populations. Together, this positions CellTag Indexing as a broadly applicable tool, easily deployed in cell culture- and transplantation-based assays, that is compatible across different single-cell modalities.

## Results

### Genetic labeling of biological samples via CellTag Indexing

**Fig. 1:**
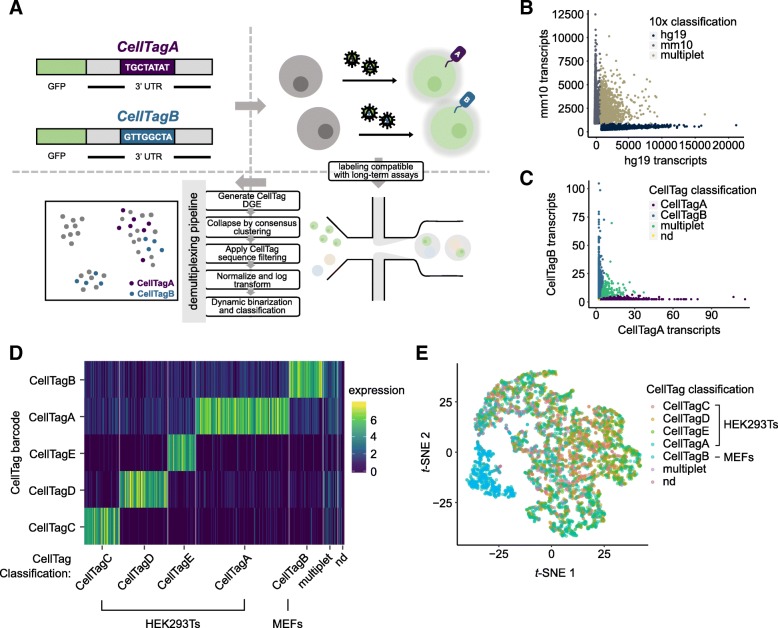
Validation of CellTag Indexing for genetic labeling of biological samples. **a** Schematic of CellTag Indexing. CellTag barcodes are positioned in the 3′ UTR of a lentiviral GFP construct with a SV40 polyadenylation signal. Barcoded viruses produced from CellTag constructs are used to transduce the cells to be “tagged.” Tagged cells can then be pooled for single-cell profiling. Prior to analysis, cell identity is demultiplexed by our classifier pipeline: A CellTag digital gene expression (DGE) matrix is generated by extracting and counting CellTag sequences for each cell; the DGE is then collapsed by consensus clustering of the detected CellTags; after filtering and log normalization, the DGE is processed by dynamic binarization and classification. Classification results can be visualized as metadata overlaying single transcriptomes projected onto reduced dimensions. **b** Scatter plot of 18,159 transcriptomes from the 2-tag species mixing experiment, classified by 10x Genomics Cell Ranger pipeline into 9357 single human cells, 7456 single mouse cells, and 1346 multiplets based on alignment to the custom hg19-mm10 reference genome. **c** Scatter plot of 18,159 transcriptomes from the 2-tag species mixing experiment, demultiplexed by CellTag Indexing into 7510 human cells (CellTagA), 6397 mouse cells (CellTagB), 1040 multiplets, and 3212 non-determined cells. **d** Log-normalized CellTag expression of the 4673 transcriptomes from the 5-tag species mixing experiment, demultiplexed into their respective sample identity on the *x*-axis; CellTag barcodes, *y*-axis. **e** Transcriptomes from the 5-tag species mixing experiment projected onto reduced dimensions by *t*-SNE, visualized with CellTag classification. CellTagC, CellTagD, CellTagE, and CellTagA label HEK293Ts; CellTagB labels MEFs

Here, we describe our lentiviral CellTag toolbox for labeling cells with transcribed DNA barcodes, acting as cell/sample identifiers that can be easily recovered from single transcriptomes. CellTag Indexing is based on the integration of defined 8-nt barcodes (CellTags), delivered via lentivirus. In this design, CellTags are positioned in the 3′ UTR of the green fluorescent protein (GFP) gene, followed by an SV40 polyadenylation signal sequence (Fig. 1a). Lentivirus carrying a defined CellTag is used to transduce and genetically label a sample, where GFP is included in this design to enable straightforward quantification of transduction efficiency. This results in the high expression of heritable, polyadenylated CellTag transcripts that are efficiently captured in standard single-cell library preparation pipelines, allowing for the demultiplexing of original sample identity in downstream analysis. We previously demonstrated the efficacy of this approach to label cells with combinations of random CellTags to support lineage tracing in cell fate reprogramming [18]. While this is a powerful approach to track clonally related cells, it requires more complex experimental design and significant computational analysis. Furthermore, only ~ 50% of labeled cells can be tracked via this method; while this supports high-confidence lineage reconstruction, it is not suited to high-efficiency cell labeling for the purpose of sample multiplexing. Our goal here was to expand the utility of CellTagging to support sample multiplexing.

First, to ensure that CellTag Indexing does not perturb cell physiology, we tested the impact of labeling on a well-characterized lineage reprogramming system, B cell to induced macrophage reprogramming [19]. We cultured HAFTL pre-B cells and induced reprogramming to macrophage fate with β-estradiol, as previously described [19]. One biological replicate was transduced with CellTag lentivirus, while an independent control replicate, cultured in parallel, was not transduced (Additional file 1: Figure S1A). After 72 h of reprogramming, the two induced macrophage samples were independently processed for sequencing, along with a sample of the original, untransduced B cells. This yielded 1310 CellTagged transcriptomes, 2849 control transcriptomes, and 972 B cell transcriptomes. We detected a median of 6 CellTag transcripts per cell in CellTagged transcriptomes (CellTags were detected in every cell of this sample) and 0 in control transcriptomes (Additional file 1: Figure S1B). Clustering and visualization [5, 20] of CellTagged and control macrophage transcriptomes are interspersed with no independent clustering observed, with both clustering separately from B cells (Additional file 1: Figure S1C&D). Additionally, CellTagged and control induced macrophages exhibit comparable upregulation of macrophage marker expression, accompanied by similar levels of B cell marker downregulation (Additional file 1: Figure S1E). Genome-wide comparison of gene expression of the two samples shows a strong linear association with an *R*^2^ value of 0.98 (Additional file 1: Figure S1F), confirming that CellTag Indexing does not perturb cell identity or physiology. This is in agreement with our previous study showing that transduction with a random CellTag library does not influence cell behavior [18].

### Species mixing of genetically distinct cells validates CellTag-based multiplexing

To assess the efficacy of CellTag-based multiplexing, we applied it to “species mixing,” an experiment commonly performed to estimate cell co-encapsulation rates in droplet-based scRNA-seq [21]. In this experiment, one sample of human HEK293T cells was labeled with CellTag Index A (CellTagA), and one sample of mouse embryonic fibroblasts (MEFs) was labeled with CellTag Index B (CellTagB), for 24–48 h. Transduction efficiency was visualized by measuring the percentage of GFP-positive cells (~ 90%, Additional file 1: Figure S2A). Labeled cells were pooled, in equal proportions, and processed together for single-cell library preparation and sequencing, yielding a total of 18,159 transcriptomes, with 9357 single human cells (aligning predominantly to the hg19 genome), 7456 single mouse cells (aligning predominantly to the mm10 genome), and 1346 multiplets as classified by 10x Genomics’ Cell Ranger pipeline, based on alignment to the custom hg19-mm10 reference genome (Fig. 1b and Additional file 1: Figure S2B). For the purpose of validation, we take this classification result as a benchmark for comparison. To assign sample identity based on CellTag Index expression, we developed a novel demultiplexing algorithm (https://github.com/morris-lab/CellTag-Classifier) [22] that examines the expression distribution of each CellTag Index, followed by a dynamic binarization step to assess each CellTag Index signal on an individual cell basis (Fig. 1a and Additional file 1: Figure S2C; see the “Methods” section). With this method, we demultiplexed the pooled transcriptomes into 7510 human cells (CellTagA), 6397 mouse cells (CellTagB), 1040 multiplets, and 3212 non-determined cells (Fig. 1c and Additional file 1: Figure S2D). Overall, our algorithm successfully classified, or demultiplexed, 82.3% of all transcriptomes. The presence of non-determined cells is likely due to cells that did not receive sufficient dosage of virus during CellTag Index transduction. This can be enhanced by increasing virus multiplicity of infection (MOI) and visualizing the percentage of GFP-expressing cells prior to sequencing, as demonstrated below. For the purpose of benchmarking, we removed the 3212 non-determined cells for comparison with the 10x-based classification (Additional file 1: Figure S2E&F). Using Cohen’s kappa as a measure of agreement between independent observations, we calculated a kappa of 0.814 (Additional file 1: Figure S2G), suggesting that our demultiplexing is in strong agreement with the orthogonal 10x-based classification. Furthermore, cells designated as multiplets by both 10x and CellTagging demonstrate a clear increase in the mean numbers of transcripts per cell (Additional file 1: Figure S2H&I), suggesting they do indeed represent multiplets.

To demonstrate the efficacy of CellTag Indexing for multiplexing several biological samples in one experiment, we conducted additional validation where four samples of HEK293Ts and one sample of MEFs were transduced with five different predefined CellTag Indexes (HEK293Ts: CellTags C, D, E, and A; MEFs: CellTag B). A total of 4673 cells were sequenced, with an inferred doublet rate of 3.6% (see the “Methods” section). Overall, CellTag expression is detected in 99.2% of all cells, reflecting the improved tagging efficiency from an increased MOI. We demultiplexed the transcriptomes as above, including an additional step to resolve misclassified multiplets (Additional file 1: Figure S2C; see the “Methods” section). Overall, 4558 out of 4673 transcriptomes, or 97.5% of all transcriptomes, were successfully classified (Additional file 2: Figure S3A). Visualization of the classified transcriptomes by heatmap of CellTag barcode expression (Fig. 1d) and by dimension reduction (Fig. 1e, Additional file 2: Figure S3B&C) demonstrates clear segregation between species, suggesting that CellTag Indexing can be used to reliably multiplex numerous samples.

### CellTag multiplexing enables long-term tracking of cell potential in an in vivo competitive transplant model

Current multiplexing methods are based on transient transfection or temporary molecular interactions with the cell or nucleus surface [7–12]. Although relative to CellTag Indexing, this offers faster labeling of cells, it does not support long-term labeling. Here, the unique advantage of CellTag-based multiplexing is that the label is heritable, as a result of stable integration into the cell genome, and can persist for many weeks as we have shown previously [18]. This creates opportunities for the longitudinal analysis of cell behavior over an extended period. Moreover, since experimental groups can be tagged, mixed, and tracked within the same biological replicate, unwanted biological and technical variation is minimized. To explore this application of CellTag multiplexing, we applied the method to assess rates of cell engraftment and intestinal differentiation potential in an in vivo competitive transplant model.

We previously reported that MEFs can be directly reprogrammed, via forced expression of transcription factors, into progenitor-like cells that possess both hepatic and intestinal potential [23, 24]. We demonstrated that these cells, named induced endoderm progenitors (iEPs), are able to functionally engraft a mouse model of induced colitis [24]. Prior to transplant, iEPs possess weak hepatic and intestinal identity, still partially resembling the fibroblasts they originated from. Twelve days after transplant into the mouse large intestine, iEPs more closely resemble differentiated intestine [24]. However, in this study, cell identity was assessed via bulk expression analysis that cannot distinguish between different intestinal cell types. Therefore, the mechanism of engraftment and differentiation potential of cells reprogrammed to iEPs remained to be characterized.

Our recent single-cell lineage tracing of fibroblast to iEP reprogramming revealed that this lineage conversion comprises two distinct trajectories: one path successfully reprogramming to iEPs, and an alternate path characterized by progression into a “dead-end” state, where fibroblast identity is re-established [18]. The transition along the successful reprogramming trajectory is accompanied by upregulation of genes such as *Apoa1* and *Cdh1* (E-cadherin). We hypothesized that the Apoa1^High^Ecad^High^ iEP cells constitute the subpopulation responsible for our previously observed colon engraftment [24]. In this context, CellTag Indexing is well-suited for tracking and quantifying reprogrammed and dead-end cell differentiation potential as the barcodes are stably integrated and heritable, making it possible to label cells for long-term tracing transplantation experiments.

**Fig. 2:**
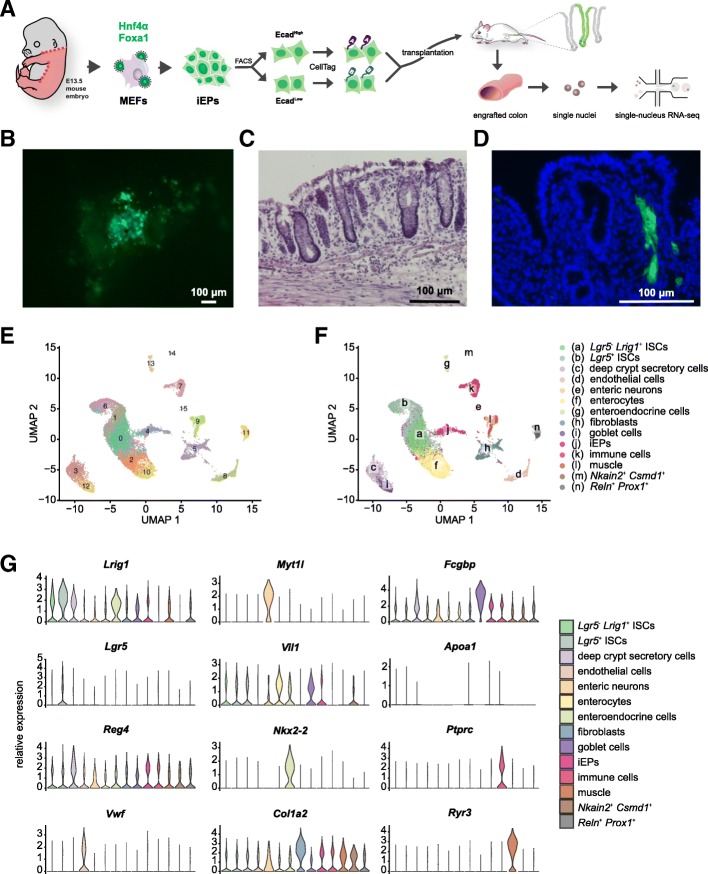
CellTag Indexing for long-term tracking of cells demonstrated in a competitive transplant experiment. **A** Schematic of iEP generation and enriched into Ecad^High^ and Ecad^Low^ populations by FACS, labeled with CellTagA and CellTagB respectively, pooled in equal proportions and transplanted into a mouse model of colonic injury. Engrafted colon is then processed for single-nucleus RNA-seq. **B** Fluorescent microscopic images of the lumen of the engrafted colon, showing patches of GFP^+^ iEPs. Scale bar, 100 μm. **C** H&E-stained section of the engrafted colon showing normal intestinal architecture with evidence of epithelial injury. Scale bar, 100 μm. **D** DAPI-stained section of the engrafted colon showing GFP^+^ iEPs in the mucosa. Scale bar, 100 μm. **E** Transcriptomes from three post-engraftment colon tissues sequenced and analyzed, visualized by UMAP, revealing 16 clusters. **F** Annotation of the 16 clusters into (a) *Lgr5*^−^ *Lrig1*^+^ intestinal stem cells (ISCs), (b) *Lgr5*^+^ ISCs, (c) deep crypt secretory cells, (d) endothelial cells, (e) enteric neurons, (f) enterocytes, (g) enteroendocrine cells, (h) fibroblasts, (i) goblet cells, (j) iEPs, (k) immune cells, (i) muscle, (m) *Nkain2*^+^ *Csmd1*^+^ cells, and (n) *Reln*^+^ *Prox1*^+^ cells. **G** Marker expression in annotated cell types

To test our hypothesis that the Apoa1^High^Ecad^High^ iEP subpopulation harbors intestinal engraftment and differentiation potential, we first enriched Ecad^High^ and Ecad^Low^ iEP populations using fluorescence-activated cell sorting (FACS). Functional assays confirmed that Ecad^High^ iEPs express significantly higher levels of *Apoa1* and *Cdh1*, form larger colonies of reprogrammed iEPs in culture, and retain their Ecad^High^ phenotype, relative to their Ecad^Low^ counterparts (Additional file 2: Figure S4A-C). We then labeled sorted Ecad^High^ iEPs with CellTagA and Ecad^Low^ iEPs with CellTagB, followed by pooling in equal proportions and transplant into a modified mouse model of colonic mucosal injury [25] (Fig. 2a). Seven days following transplantation, mice were euthanized and dissected, and the engrafted colons collected for histology and single-nucleus RNA sequencing. Microscopic examination of the engrafted tissue reveals iEP engraftment in discrete patches, located by their GFP expression (Fig. 2b). Histology of the cryosectioned engrafted colon shows the expected tissue architecture with evidence of epithelial injury (Fig. 2c), occasional submucosal iEPs (Fig. 2d), and occasional aggregates of iEPs sitting atop of the damaged epithelium (Additional file 2: Figure S4D).

Most intestinal cell recovery protocols focus on the harvest of the epithelium, neglecting many other cell types that constitute the intestine. Given the range of engraftment phenotypes observed in our above histology analyses, we considered that iEPs may also differentiate towards non-epithelial cell types. Thus, to capture the full spectrum of intestinal cell identities, we opted to use whole tissue single-nucleus extraction, over epithelial isolation and digestion, to process the engrafted colon for RNA sequencing. Indeed, single-nucleus RNA sequencing (snRNA-seq) from three colon samples, post-engraftment, followed by Uniform Manifold Approximation and Projection (UMAP)-based visualization [20] revealed 16 clusters (Fig. 2e), corresponding to a range of different intestinal epithelial cell types. This included intestinal stem cells (ISCs), enterocytes, deep crypt secretory cells, goblet cells, enteroendocrine cells, and non-epithelial cell types (endothelial cells, muscle, enteric neurons, immune cells, fibroblasts) (Fig. 2f). To our knowledge, this is the first dataset of such that profiles large intestinal cell types beyond the epithelium. Known intestinal markers are observed such as *Lgr5*, *Lrig1*, and *Smoc2* in ISCs [26–29]; *Reg4* in deep crypt secretory cells [30]; *Myt1l*, *Asic2*, and *Syt1* in enteric neurons [31–34]; *Vil1*, *Plac8*, and *Krt20* in enterocytes [35–37]; *Nkx2-2*, *Chga*, and *Tph1* in enteroendocrine cells [38, 39]; and *Fcgbp*, *Muc2*, and *Clca1* in goblet cells [40, 41] (Fig. 2g, Additional file 2: Figure S5C-E).

Upon further analysis and literature review, we annotated the ISCs into two populations, *Lgr5*^*+*^ ISCs (clusters 1 and 6) and *Lgr5*^*−*^ *Lrig1*^*+*^ ISCs (cluster 0), based on distinct patterns of marker expression (Additional file 2: Figure S5C). *Lrig1*, a transmembrane negative regulator of ErbB signaling [42], is purported to mark a class of ISCs that are phenotypically distinct from *Lgr5*^*+*^ stem cells in the intestine [27, 28], with additional roles in stem cells of the gastric epithelium [43] and the epidermis [44–46]. *Lgr5*^*+*^ ISCs, located in clusters 1 and 6 in this dataset, express high levels of established intestinal stem cell markers *Lgr5* and *Smoc2*, as well as *Lrig1* (Fig. 2g, Additional file 2: Figure S5C-E). In contrast to *Lgr5*, *Lrig1* is more widely expressed, with moderate levels of expression extending into cluster 0, where *Lgr5* expression is absent (Additional file 2: Figure S5C). This is consistent with two independent studies in the small intestine and colon, where *Lrig1* was expressed in many crypt cells, while the highest levels of *Lrig1* expression were observed in *Lgr5*^*+*^ stem cells [27, 28]. Loss of Lrig1 caused crypt expansion in Lrig1-knockout animals, and three-dimensional intestinal spheres derived from Lrig1-knockout animals matured into budding organoids in culture without exogenous ErbB ligands in contrast to wild-type samples [27]. Intriguingly, *Lrig1* was shown to mark a population of ISCs that expand and repopulate the colonic crypt upon tissue damage [28], although a distinction was not made regarding whether this could be due to the subpopulation of *Lrig1*^*+*^ cells that are also *Lgr5*^*+*^.

Of note, two clusters that remain unannotated (cluster 11, enriched for *Reln* and *Prox1*; cluster 14, enriched for *Nkain2* and *Csmd1*) may represent rare or previously unidentified cell types (Additional file 2: Figure S5D). For example, *Reln* and *Prox1* are known for their roles in neuronal migration [47, 48] and neurogenesis [49, 50]; we, therefore, speculate that they may mark a peripheral neuronal cell type in cluster 11.

### Colon engrafted iEPs transition through an intestinal stem cell state

**Fig. 3:**
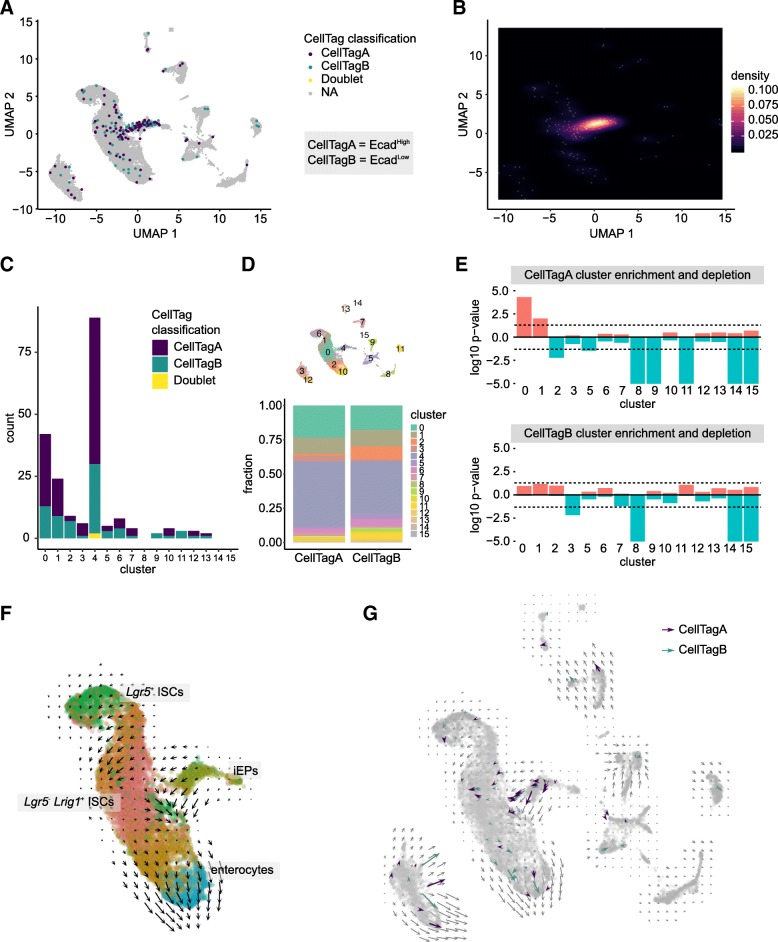
CellTag Indexing revealed iEP engraftment and transition through an intestinal stem cell fate. **a** CellTags identified engrafted iEPs enriched in cluster 4 (early engraftment iEPs) and the main intestinal epithelial clusters. **b** Density heatmap confirms enrichment of CellTagged cells in the early engraftment iEP cluster and the main intestinal epithelial cell clusters. **c**, **d** Stacked bar plots of CellTagged cells show enrichment in clusters 0, 1, and 4. **e** Permutation test of cluster enrichment or depletion for each CellTag in intestinal clusters show statistically significant enrichment of Ecad^High^/CellTagA cells in cluster 0 (Lgr5^−^ Lrig1^+^ ISCs, *p* = 4.03 × 10^− 5^) and cluster 1 (Lgr5^+^ ISCs, *p* = 9.83 × 10^− 3^). *y*-axis, negative log10 of *p* value for cluster enrichment, log10 of *p* value for cluster depletion. Dotted lines correspond to a *p* value of 0.05. **f** RNA velocity analysis shows velocity vectors from iEPs towards *Lgr5*^−^ *Lrig1*^+^ ISCs and from the ISC clusters towards the differentiated enterocyte clusters. **g** Subset of velocity vectors of CellTagged cells confirm transcriptional kinetics of engrafted iEPs in the direction towards intestinal stem cells

To identify iEPs within the single-nucleus landscape of the engrafted colon, we extracted and processed CellTag Indexes across all single transcriptomes. Both CellTagA and CellTagB barcodes were detected in all three post-engraftment samples (Additional file 1: Figure S6A), with clear expression differences between tags (Additional file 2: Figure S6B). Projecting CellTagged iEPs onto the UMAP plot revealed their enrichment in cluster 4 (Fig. 3a, b), while a moderate number of CellTagged cells are found in intestinal epithelial clusters such as cluster 0 (*Lgr5*^*−*^ *Lrig1*^*+*^ ISCs) and cluster 1 (*Lgr5*^*+*^ ISCs), expressing ISC markers *Lgr5* and *Lrig1* (Fig. 3c, d, Additional file 2: Figure S6C).

Engrafted tissue was harvested in early stages of intestinal regeneration, with the epithelium still undergoing active repair. We chose this time point in an effort to understand the mechanism of iEP engraftment. Indeed, in line with this early regeneration period, cluster 4 likely represents cells in the early stages of engraftment and repair, characterized by expression of both intestinal and mesenchymal markers (Additional file 2: Figure S5D&E). Notably, Grip1, an adaptor protein implicated in maintaining the epidermal-dermal junction via the Fras1/Frem1 complex [51, 52], is among the list of marker genes for cluster 4, suggesting that cluster 4 might represent an iEP engraftment mechanism via adhesion to the basement membrane. We next focused on the proportions of fully reprogrammed Ecad^High^ iEPs (labeled by CellTagA) and dead-end Ecad^Low^ iEPs (labeled by CellTagB) engrafting the intestine. We found that 0.687% ± 0.214% of engrafted cells were derived from reprogrammed iEPs whereas 0.413% ± 0.113% of engrafted cells were derived from dead-end iEPs (*p* = 0.06; Additional file 2: Figure S6D). This low percentage was expected given that we aimed to capture a broad range of intestinal engraftment to provide an unbiased assessment of engraftment.

In our previous study, we observed that iEPs are capable of long-term (7 weeks post-transplant), functional engraftment, where entire crypts are repopulated by iEP-derived cells [24]. At that time, we speculated that iEPs transition through an intestinal stem cell state to support long-term engraftment. Here, considering our hypothesis that fully reprogrammed Ecad^High^ iEPs are responsible for this long-term engraftment, we performed a randomized test that we previously developed to assign statistical significance in cluster occupancy [18]. Here, we applied this approach to determine whether reprogrammed and dead-end iEPs were more likely to associate with any particular cluster of intestinal cells. We did not include cluster 4 in this analysis as the colonic epithelium is in the early stages of regeneration, where we consider cells in this cluster to be superficially attached, and not all these cells will eventually integrate into the recovered epithelium. Our randomized test revealed that reprogrammed Ecad^High^/CellTagA cells are significantly more likely to occupy cluster 0 (*Lgr5*^*−*^ *Lrig1*^*+*^ ISCs, *p* = 4.03 × 10^− 5^) and cluster 1 (*Lgr5*^*+*^ ISCs, *p* = 9.83 × 10^− 3^), while CellTagged reprogrammed and dead-end populations are depleted from non-epithelial cell clusters (Fig. 3e). Together, this suggests that Ecad^High^/CellTagA cells integrate into the regenerating epithelium via an intestinal stem cell intermediate. Expression of the ISC markers *Lgr5* and *Lrig1* in engrafted iEPs supports this observation (Additional file 2: Figure S6C). As reported previously, *Lrig1*^*+*^ ISCs expand and repopulate the colonic crypt upon tissue damage [28], pointing to a potential mechanism of long-term iEP engraftment in the mouse colon.

To further investigate engraftment mechanics, we conducted RNA velocity analysis [53] to reveal the transcriptional kinetics of engrafting iEPs. We reasoned that if these iEPs were differentiating towards intestinal lineages, then transcript kinetics from early iEP engraftment cluster, cluster 4, should show velocity vectors towards annotated intestinal clusters. Indeed, RNA velocities projected onto the UMAP clusters show cluster 4 velocities towards the main intestinal clusters (Fig. 3f, Additional file 2: Figure S6F&G). Specifically, velocity vectors from the subset of CellTagged cells show vectors originating from cluster 4 towards cluster 0, and from the intestinal stem cell pole of the main intestinal clusters towards the more differentiated pole of enterocytes (Fig. 3g). Taken together, here, we have demonstrated the utility of CellTag Indexing to multiplex Ecad^High^ and Ecad^Low^ iEPs for transplantation into the mouse large intestine, suggesting that iEPs transition through a *Lgr5*^*+*^ and/or *Lrig1*^*+*^ stem cell state to engraft and repopulate the colonic epithelium, resolving speculation about their engraftment route. Our findings are consistent with previous reports of iEP differentiation potential and position CellTag Indexing as a powerful long-term tracking and multiplexing tool for scRNA-seq.

## Discussion

Here, we present a broadly applicable toolbox, CellTag Indexing, to label biological samples for single-cell analysis, where each sample is genetically tagged with a predefined lentiviral GFP barcode to mark its sample identity. We demonstrate that CellTag Indexing does not perturb cell physiology, and validate the utility of our multiplexing approach via species mixing, showing that it can be used to accurately multiplex samples for scRNA-seq, with subsequent demultiplexing at high efficiency. We showcase the unique feature of this heritable labeling approach, by tracking cells in a competitive in vivo transplant setting, revealing reprogrammed cell potential and mechanisms of engraftment while providing internal controls to mitigate both biological and technical batch effects. CellTag multiplexing is complementary to current strategies based on transient cell surface interactions for labeling cells immediately prior to scRNA-seq, yet unique in that CellTag barcodes are stably integrated and heritable through cell division. The flexible timing of lentiviral barcode transduction, coupled with stable barcode expression, makes our system uniquely suitable for long-term tracing experiments and transplant models where temporary tags would not be retained.

CellTag Indexing offers the advantages of minimized technical variation by experimental design, the ability to multiplex biological samples for competitive transplant, broad compatibility with various cell types and single-cell technologies, long-term barcode expression, streamlined workflow and library preparation, reduced sequencing cost, and straightforward demultiplexing. CellTag Indexing is designed for broad applications; its use of lentivirus as a labeling method represents a commonly used and accessible biological tool with minimal setup costs and reagent requirements. As lentivirus can transduce both dividing and non-dividing cells, CellTag barcodes can be introduced into a wide variety of cell types. In terms of estimating labeling efficiency, CellTag Indexing conveniently utilizes GFP as a barcode carrier, which can act as a visual readout for transduction efficiency. Generally, CellTag transcripts are abundantly expressed and can be optionally amplified during library preparation to further increase the detection rate.

Importantly, CellTag transcripts can be recovered from the nucleus, extending this approach to single-nucleus RNA sequencing. Furthermore, cells labeled with CellTag Indexes can be cultured and used in experiments prior to collection for sequencing, for example in the competitive transplant assay we demonstrated here where tagged samples act as internal controls for each other to minimize unwanted biological variation. This is complementary to existing labeling methods that utilize cell/nuclear surface chemistry or transient transfection for temporary tagging [7–12], where the labels would be progressively lost in vitro and in vivo. Additionally, as a future application, we expect that CellTag multiplexing will be compatible with single-genome-based assays such as single-cell ATAC-seq. In summary, CellTag Indexing is a broadly applicable tool complementary to existing methods for cell multiplexing and tracking, providing a diverse panel of experimental and analytical strategies.

## Methods

### Cell culture

Mouse embryonic fibroblasts were derived from the C57BL/6 J strain (The Jackson Laboratory 000664). HEK293T and mouse embryonic fibroblasts were cultured in Dulbecco’s modified Eagle medium (Gibco) supplemented with 10% fetal bovine serum (Gibco), 1% penicillin/streptomycin (Gibco), and 55 μM 2-mercaptoethanol (Gibco). HAFTL pre-B cells were cultured in RPMI1640 without phenol red (Lonza) supplemented with 10% charcoal/dextran-treated FBS (Hyclone) and 55 μM 2-mercaptoethanol (Gibco) [19].

### Generation of iEPs

Mouse embryonic fibroblasts were converted to iEPs as previously described [23, 24]. Briefly, fibroblasts were prepared from E13.5 embryos, cultured on gelatin, and serially transduced every 12 h with Hnf4α-t2a-Foxa1 retrovirus for five times over the course of 3 days, followed by culture on collagen in hepato-medium, which is DMEM:F-12 (Gibco) supplemented with 10% FBS, 1% penicillin/streptomycin, 55 μM 2-mercaptoethanol, 10 mM nicotinamide (Sigma-Aldrich), 100 nM dexamethasone (Sigma-Aldrich), 1 μg/mL insulin (Sigma-Aldrich), and 20 ng/ml epidermal growth factor (Sigma-Aldrich).

### CellTag barcodes

CellTag lentiviral constructs were generated by introducing an 8-bp variable region into the 3′ UTR of GFP in the pSmal plasmid [54] using a gBlock gene fragment (Integrated DNA Technologies) and megaprimer insertion (https://www.addgene.org/pooled-library/morris-lab-celltag/). Individual clones were picked and Sanger sequenced to generate predefined barcodes. The specific CellTag barcodes used in this manuscript are TGCTATAT (CellTagA), GTTGGCTA (CellTagB), AGTTTAGG (CellTagC), GGTTCACA (CellTagD), and TAGAAAGC (CellTagE). These constructs are available from Addgene: https://www.addgene.org/browse/article/28197603/.

### Lenti- and retrovirus production

Lentiviruses were produced by transfecting HEK293T cells with lentiviral pSMAL vector and packing plasmids pCMV-dR8.2 dvpr (Addgene plasmid 8455) and pCMV-VSV-G (Addgene plasmid 8454) using X-tremeGENE 9 (Sigma-Aldrich). Viruses were collected 48 and 72 h after transfection. Retroviruses were similarly produced, with retroviral pGCDNSam vector and packaging plasmid pCL-Eco (Imgenex).

### CellTag transduction

CellTag virus-containing supernatant collected from virus-producing HEK293T cells was kept at 4 °C and used within 1 week. Prior to transduction, protamine sulfate (Sigma-Aldrich) was added to the viral solution to a final concentration of 4 μg/ml. Cells were aspirated of media, and the CellTag virus was added to the cells for a 24-h transduction period. This transduction was repeated as needed, for a total of 48 h for HEK293T cells and 72 h for MEFs in the 5-tag species mixing experiment and 72 h for iEPs.

### Immunostaining and quantification

Transduced HEK293T and MEFs were cultured on a four-chamber culture slide (Falcon) for 24 h prior to fixation in 4% paraformaldehyde and staining in 300 nM DAPI in PBS. The slide was then mounted in ProLong Gold Antifade Mountant (Invitrogen). Images were acquired on a Nikon eclipse Ts2 inverted microscope. For automatic quantification, images of CellTagged HEK 293T and MEF were processed with a custom python script to count GFP-positive/negative cells. The proportion of GFP-positive cells was calculated from DAPI and GFP images. First, DAPI images were transformed into binary images by thresholding fluorescent signal. The threshold values were determined by the Otsu method. The binary nucleus image was processed by watershed segmentation to separate individual cell areas completely. Inappropriately sized objects were filtered to remove noise and doublet cells. The intensity of the GFP signal per individual cell area was then quantified to distinguish between GFP-positive cells and GFP-negative cells. These processes were run with Python 3.6.1 and its libraries: scikit-image 0.13.0, numpy 1.12.1, matplotlib 2.0.2, seaborn 0.8.1, jupyter 1.0.0.

### Mouse model of colonic mucosal injury

Using a previously described procedure [25], we generated colonic epithelial injury with modifications as followed: C57BL/6 mice were anesthetized with inhaled isoflurane. A custom-made syringe catheter (consisted of 3-ml syringe (BD #309657), Luer lock 26-gauge 1/2″ dispensing needle (GraingerChoice #5FVG9), and polyethylene tubing (Scientific Commodities, #BB31695-PE/2) cut to approximate 5 cm in length and affixed to the needle) was used to deliver approximately 1 mL of PBS enema intraluminally via the anal canal, followed by gentle abdominal massage to promote excretion of excess fecal matter. The luminal space was then filled with 0.5 mL of 500 mM EDTA/PBS using the custom syringe catheter over the course of approximately 30 s. Mechanical abrasion was performed with Proxabrush cleaners (Sunstar #872FC) dipped in 500 mM EDTA/PBS, inserted approximately 1 cm into the colon, with 30 rotational movements to gently scratch the luminal surface.

### iEP characterization and transplantation

Eight-week iEPs were stained with mouse E-cadherin-APC antibody (10 μL per one million cells, R&D Systems, FAB7481A) and sorted on a modified Beckman Coulter MoFlo into Ecad^High^ and Ecad^Low^ populations. Sorted iEPs were plated and cultured as above. Colony formation assay was performed as previously described [18]. For colon engraftment, CellTagged Ecad^High^ and Ecad^Low^ iEPs were digested into single-cell suspensions. For each mouse, 0.5 million of Ecad^High^ iEPs (CellTagA) and 0.5 million of Ecad^Low^ iEPs (CellTagB) were pooled and resuspended in 50 μL of 10% Matrigel on ice. A total of 1 million iEPs was instilled into the colonic lumen of each mouse by using the custom syringe catheter, after which the mouse was held vertically head-down for approximately 2 min to prevent immediate excretion of the infused cell suspension.

### Single-nucleus RNA-seq procedure

Single-nucleus extraction from tissue was performed as previously described [55]. Briefly, engrafted colonic tissues were finely minced with a razor then transferred to a Dounce tissue homogenizer (Kimble Chase KT885300-0002) in 2 mL of ice-cold Nuclei EZ Lysis buffer (Sigma #N-3408) supplemented with protease inhibitor (Roche #5892791001) and RNase inhibitors (Promega #N2615, Thermo Fisher Scientific #AM2696). The tissue was ground 20–30 times with the loose pestle. The homogenate was filtered through a 200-μm cell strainer (pluriSelect #43-50200) then transferred back to the Dounce homogenizer, ground with the tight pestle 10–15 times. The homogenate was incubated on ice for 5 min with an additional 2 mL of lysis buffer, then filtered through a 40-μm cell strainer (pluriSelect #43-50040) and centrifuged at 500*g* for 5 min at 4 °C. The incubation and centrifugation steps were repeated one time, followed by resuspension Nuclei Suspension Buffer (1× PBS, 1% BSA, 0.1% RNase inhibitor) and filtering through a 5-μm cell strainer (pluriSelect #43-50005). The nuclei were then loaded onto the 10x Chromium Single Cell Platform for encapsulation and barcoding.

### scRNA-seq procedure

10x Chromium Single Cell 3′ Library & Gel Bead Kit, 10x Chromium Single Cell 3′ Chip kit, and 10x Chromium i7 Multiplex kit (10x Genomics) were used according to the manufacturer’s protocols. Libraries were quantified on the Agilent 2200 TapeStation and sequenced on Illumina HiSeq 2500.

### CellTag demultiplexing

Details of the CellTag Classifier can be found on the GitHub repository (https://github.com/morris-lab/CellTag-Classifier) [22]. Briefly, the CellTag count matrix is extracted as previously described [18] (outlined at https://github.com/morris-lab/CellTagWorkflow). CellTag sequences are collapsed using Starcode with the sphere clustering algorithm [56], where CellTags with similar sequences were collapsed to the centroid CellTag. The collapsed CellTag count matrix is log-normalized, from which the most highly expressed CellTags across cells are selected. Then, a dynamic binarization method is applied to assess the existence of each CellTag in each cell, where a “0” suggests insignificant/unobservable signals and a “1” indicates a significant signal. Specifically, for each CellTag, we compute the density function *D* of its expression across all cells. Then, for each cell, we draw 1000 samples from the density functions *D* and calculate the proportion *P* of samples that are greater than or equal to the expression value being tested:

$$ P=\frac{\mathrm{II}\left(S\ge {C}_{ij}\right)}{\mathrm{Length}\ \mathrm{of}\ S} $$

where *C*_*ij*_ = expression value of CellTag *j* in cell *i* and *S* = 1000 sample drawn from the density curve of CellTag *j*, *D*_*j*_. This process is iterated for at least 50 times to make sure that the samples are representative of the overall density. The cells are then classified to their corresponding CellTag based on the proportions calculated above by finding the overall minimum in each proportion matrix. The uniqueness of the minimum does not eliminate the probability for the cell to be a multiplet. Hence, for cells with a unique minimum, we examine the pair-wise differences between the minimum tag and other tags using a baseline cutoff of 0.238 learned via benchmarking and training against orthogonal 10x classification. Finally, the number of multiplets identified from our pipeline is compared to the expected number derived from 10x Genomics’ Single Cell 3′ Reagents Kit v2 User Guide Rev. E (multiplet % = 0.0007589 × number of cells recovered + 0.0527214). If the number of multiplets exceeds the expected number, the optional multiplet checkpoint is implemented, where the proportion matrix is sorted such that the most likely multiplets are identified using a cutoff at the quantile of (1.5 × expected num/multiplet). The remaining cells are then classified to their singlet identities.

### scRNA-seq analysis

The Cell Ranger v.3.0.1 pipeline (https://support.10xgenomics.com/single-cell-gene-expression/software/downloads/latest) was used to process data generated using the 10x Chromium platform. For alignment of the single-nucleus RNA-seq data, a modified “pre-mRNA” mm10 reference was used to include reads aligned to introns. The R package Seurat [5] (version 3) was used for data processing and visualization. For the iEP dataset, we removed cells with a low number of genes detected (< 200), cells with a high number of UMI detected (> 100,000), and cells with a high proportion of UMI counts attributed to mitochondrial genes (> 20%). The filtered expression matrix was then normalized and scaled to remove unwanted sources of variation driven by the number of detected UMIs and mitochondrial gene expression. Linear dimension reduction was performed, followed by canonical correlation analysis to integrate independent biological replicates, then clustering and visualization via UMAP [20].

### Assessing cluster occupancy by randomized testing

A randomized test that we developed previously [18] was used to identify clusters that are significantly occupied by Ecad^High^/Ecad^Low^ iEPs. In brief, we calculated the proportions of CellTagA and CellTagB cells that fall into each cluster, serving as the null percentages for the two tags. In particular, let *n* be the number of cells with a CellTag. Let *s* be the number of cells without this tag. The two were then pooled together from which we drew *n* random samples without replacement for at least *(n + s)/n* times such that every possible ending group can be captured. With each sample drawn, the occupancy of *n* sampled cells in each cluster was calculated. A background proportion distribution was then generated based on this resampling result. We then used the distributions to compute the likelihood of having the null percentage or higher. Using a *p* value of < 0.05, we identified the clusters that are enriched for each CellTag. This randomized test was performed using a python script. We exclude cluster 4 in this test as it represents the early engraftment stage. Cell number tested for CellTagA equals 66. Cell number tested for CellTagB equals 46.

### RNA velocity analysis

RNA velocity was analyzed with Velocyto.py (version 0.17.17). The analysis was done according to the web instruction; http://velocyto.org/velocyto.py/. For the input of single-cell RNA-seq data, the output files of 10x Cell Ranger pipeline were used. The single-cell RNA-seq reads for each sample were converted into read-counts after distinguishing a spliced or unspliced transcript. This process was done with command line velocyto API, and final products were saved as loom files. Next, the loom files of each scRNA-seq sample were merged into a single loom file. The merged loom file was processed with velocyto python API to create the velocyto object. Then, the velocyto object was integrated with UMAP dimensional reduction data and CellTag data which were produced in the scRNA-seq analysis with Seurat and CellTag demultiplexing process. Next, the velocyto object was subjected to quality check and filtering process. Genes were filtered by the mRNA detection level (min_expr_counts = 40, min_cells_express = 30). After feature selection by a velocyto function, the data were normalized by total molecule count. Then, velocyto object was subjected to a series of final data processing process: PCA, k-nn-based imputation, velocity estimation, and shift calculation. Finally, the vectors estimated by RNA velocity were projected on the UMAP graph.

## Additional files

Additional file 1: Supplementary Figure S1 and S2 (PDF 18535 kb)

Additional file 2: Supplementary Figure S3-S6 (PDF 14243 kb)
