# Supplementary material for: CellTag Indexing: genetic barcode-based sample multiplexing for single-cell genomics
Source: Genome Biol. 2019 May 9;20:90. doi: 10.1186/s13059-019-1699-y (PMC6509836; doi:10.1186/s13059-019-1699-y)
Supplement: Supplementary file 1 — Supplementary Figure S1 and S2 (PDF 18535 kb) [file 13059_2019_1699_MOESM1_ESM.pdf]

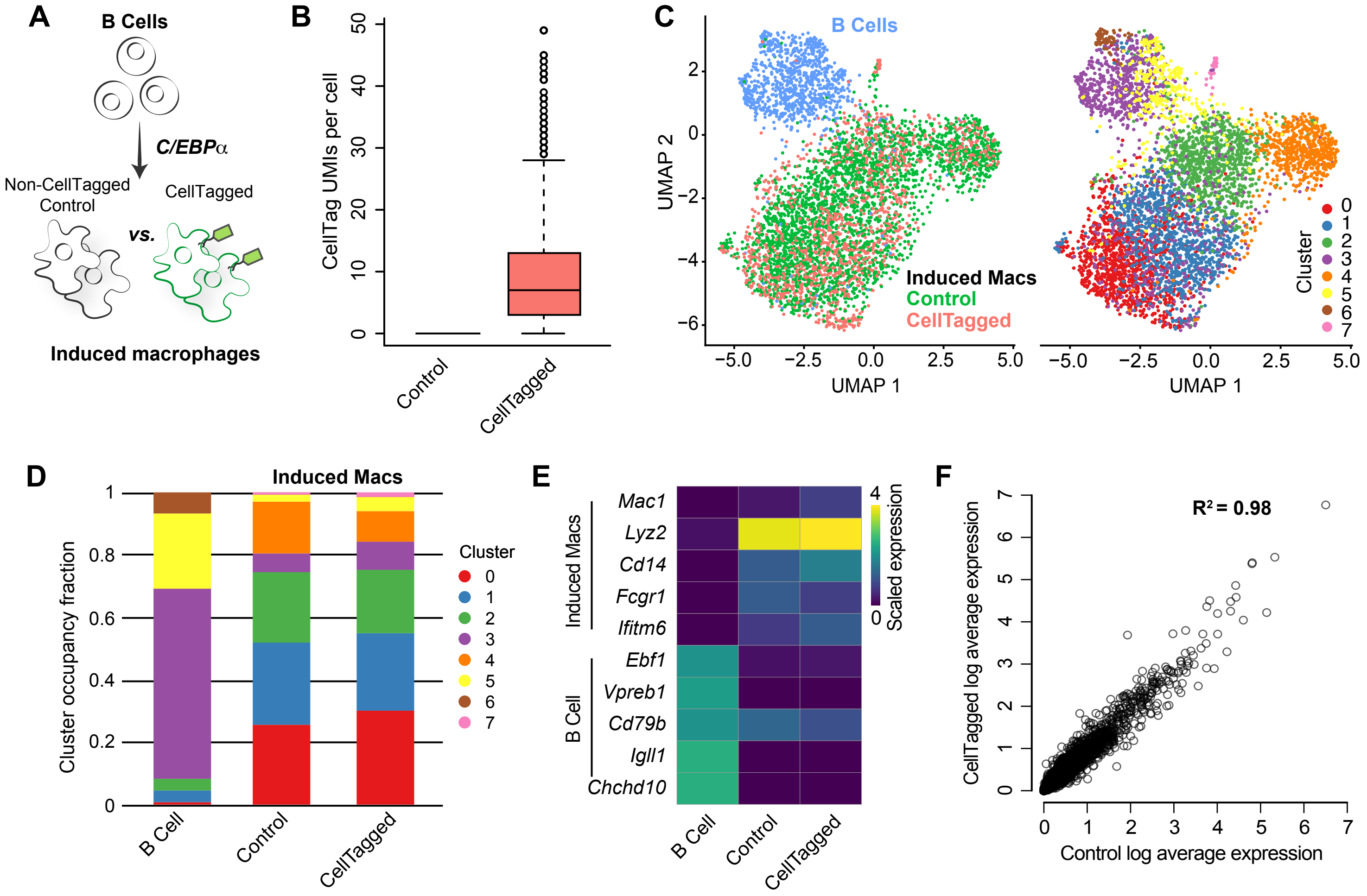
A
B Cells
C/EBPα
Non-CellTagged Control
CellTagged
vs.
Induced macrophages
B
CellTag UMIs per cell
Control
CellTagged
C
B Cells
UMAP 2
UMAP 1
Induced Macs
Control
CellTagged
Cluster
D
Induced Macs
Cluster occupancy fraction
Cluster
B Cell
Control
CellTagged
E
Induced Macs
Mac1
Lyz2
Cd14
Fcgr1
Ifitm6
B Cell
Ebf1
Vpreb1
Cd79b
Igll1
Chchd10
Scaled expression
F
R² = 0.98
CellTagged log average expression
Control log average expression

Fig. S1 CellTag does not interfere with normal cell physiology or reprogramming. **A,** Schematic of B-cell to macrophage reprogramming and CellTagging. **B,** A median of 6 CellTag transcripts are detected per cell in CellTagged transcriptomes (CellTags were detected in all cells of this sample), while none are detected in control transcriptomes. **C,** CellTagged and control macrophages transcriptomes are interspersed with no independent clustering; both cluster separately from B cells. **D,** CellTagged and control macrophages transcriptomes share indistinguishable cluster composition, both distinct from B cell transcriptomes. **D,** CellTagged and control macrophages transcriptomes have similar macrophage marker expression and downregulate B cell marker expression. **E,** Genome-wide gene expression between CellTagged and control transcriptomes are strongly correlated with an R^2 of 0.98.

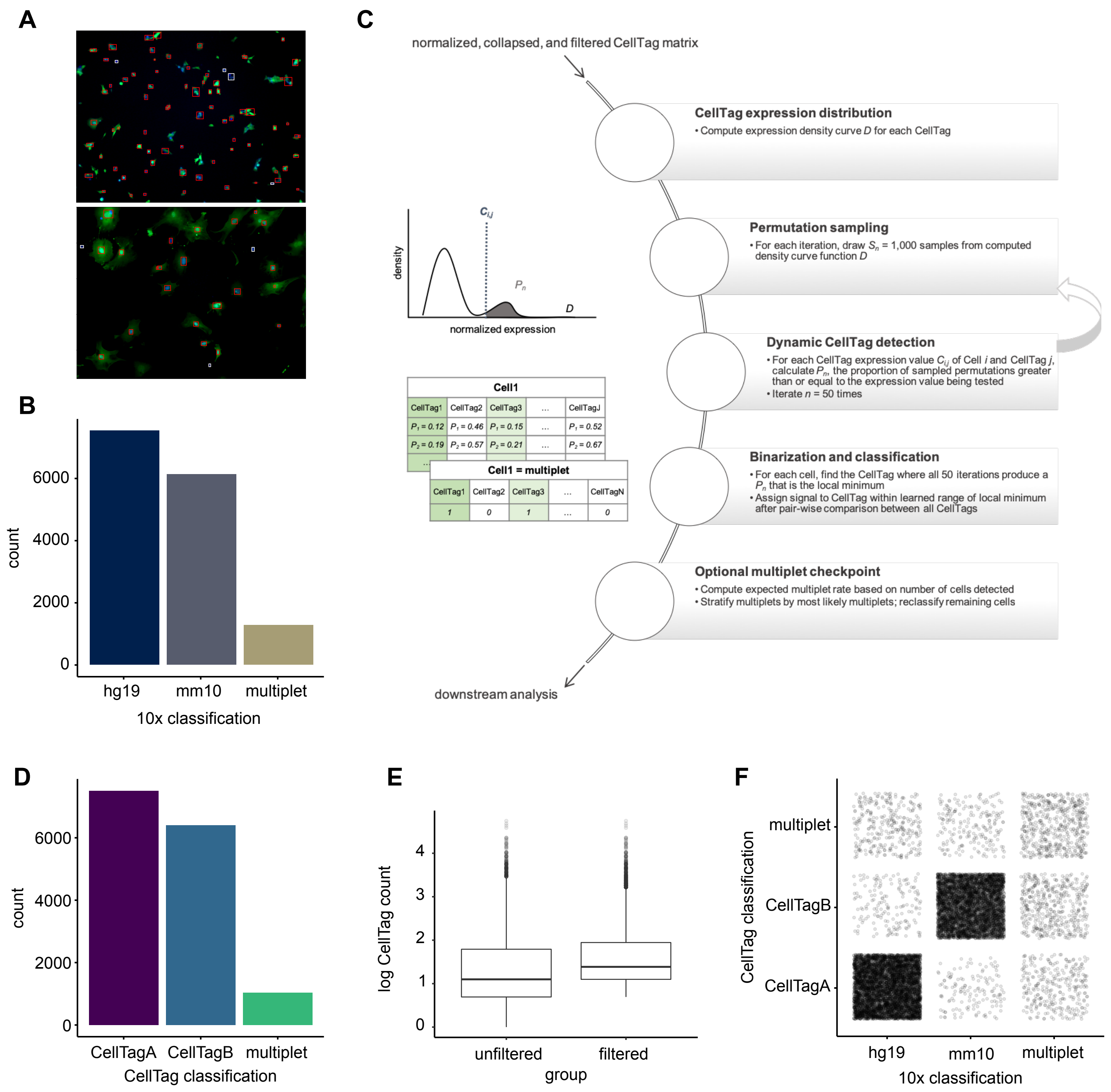

G

	CellTagA	CellTagB	multiplet
hg19	7068	162	308
mm10	142	5783	207
Multiplet	300	452	525

Cohen's Kappa for 2 Raters
(Weights: unweighted)

Subjects = 14947
Raters = 2
Kappa = 0.814
z = 120
p-value = 0

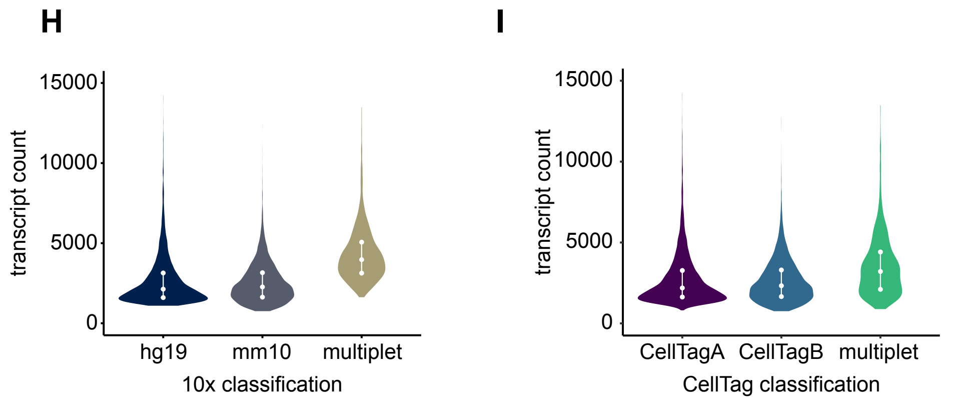

Fig. S2 CellTag classification compared with 10x-based classification the 2-tag species mixing experiment. **A,** Visualization and quantification of GFP expression in DAPI-stained HEK293Ts (top, 95%) and MEFs (bottom, 88%) transduced with CellTag virus for 24-48 hours. Red box, $DAPI^{+}$ GFP^{+}; white box, $DAPI^{+}$ GFP^{-}. **B,** 10x-based classification; excluding non-determined cells. **C,** Schematic workflow of the dynamic binarization and classification framework. **D,** CellTag classification; excluding non-determined cells. **E,** Log-normalized CellTag count before and after filtering for non-determined cells. **F,** Visual comparison of CellTag and 10x-based classification. **G,** Table comparing CellTag and 10x-based classification, benchmarked using Cohen's kappa as a measure of agreement. Unweighted Cohen's Kappa = 0.814. **H&I,** Total transcript count in different groups as classified by 10x- or CellTag-based classification. Dotted bars, first, median, and third quartiles.
