# Supplementary material for: CellTag Indexing: genetic barcode-based sample multiplexing for single-cell genomics
Source: Genome Biol. 2019 May 9;20:90. doi: 10.1186/s13059-019-1699-y (PMC6509836; doi:10.1186/s13059-019-1699-y)
Supplement: Supplementary file 2 — Supplementary Figure S3-S6 (PDF 14243 kb) [file 13059_2019_1699_MOESM2_ESM.pdf]

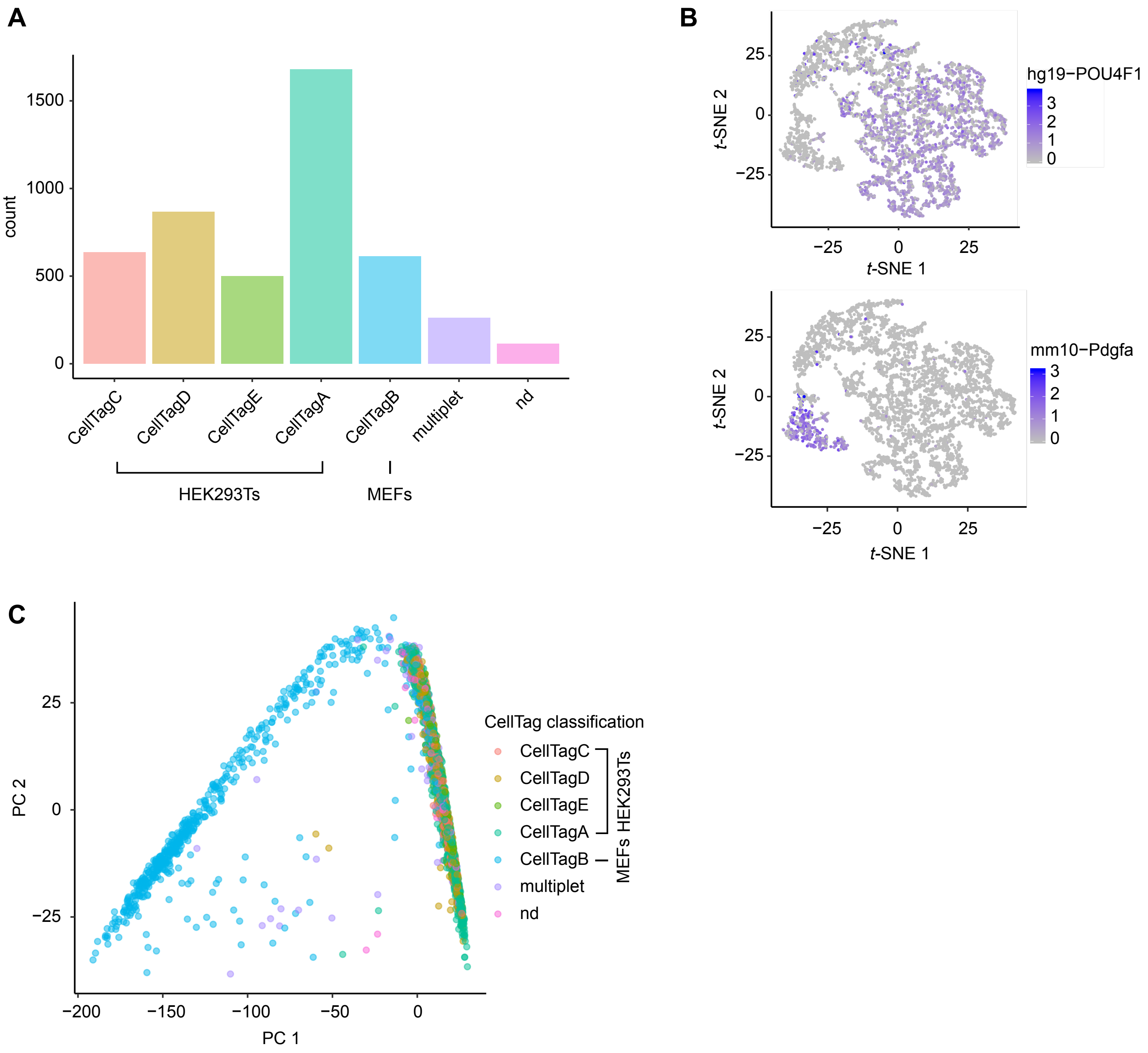

A
count
1500
1000
500
0
CellTagC
CellTagD
CellTagE
CellTagA
CellTagB
multiplet
nd
HEK293Ts
MEFs
B
t-SNE 2
t-SNE 1
25
0
−25
hg19−POU4F1
3
2
1
0
mm10−Pdgfa
C
PC 2
PC 1
−200
−150
−100
−50
CellTag classification
CellTagC
CellTagD
CellTagE
CellTagA
CellTagB
multiplet
nd
HEK293Ts
MEFs

Fig. S3 CellTag classification in 5-tag species-mixing experiment. **A,** CellTag classification of the 5-tag species mixing experiment into 637 CellTagC (HEK293T), 867 CellTagD (HEK293T), 501 CellTagE (HEK293T), 1,679 CellTagA (HEK293T), 612 CellTagB (MEF), 262 multiplet, and 115 non-determined cells. **B,** Expression pattern of HEK293T marker *POU4F1* and MEF marker *Pdgfa* in mixed transcriptomes projected onto *t*-SNE. **C,** CellTag classification visualized over transcriptomes projected onto principal component 1 (PC 1) and PC 2.

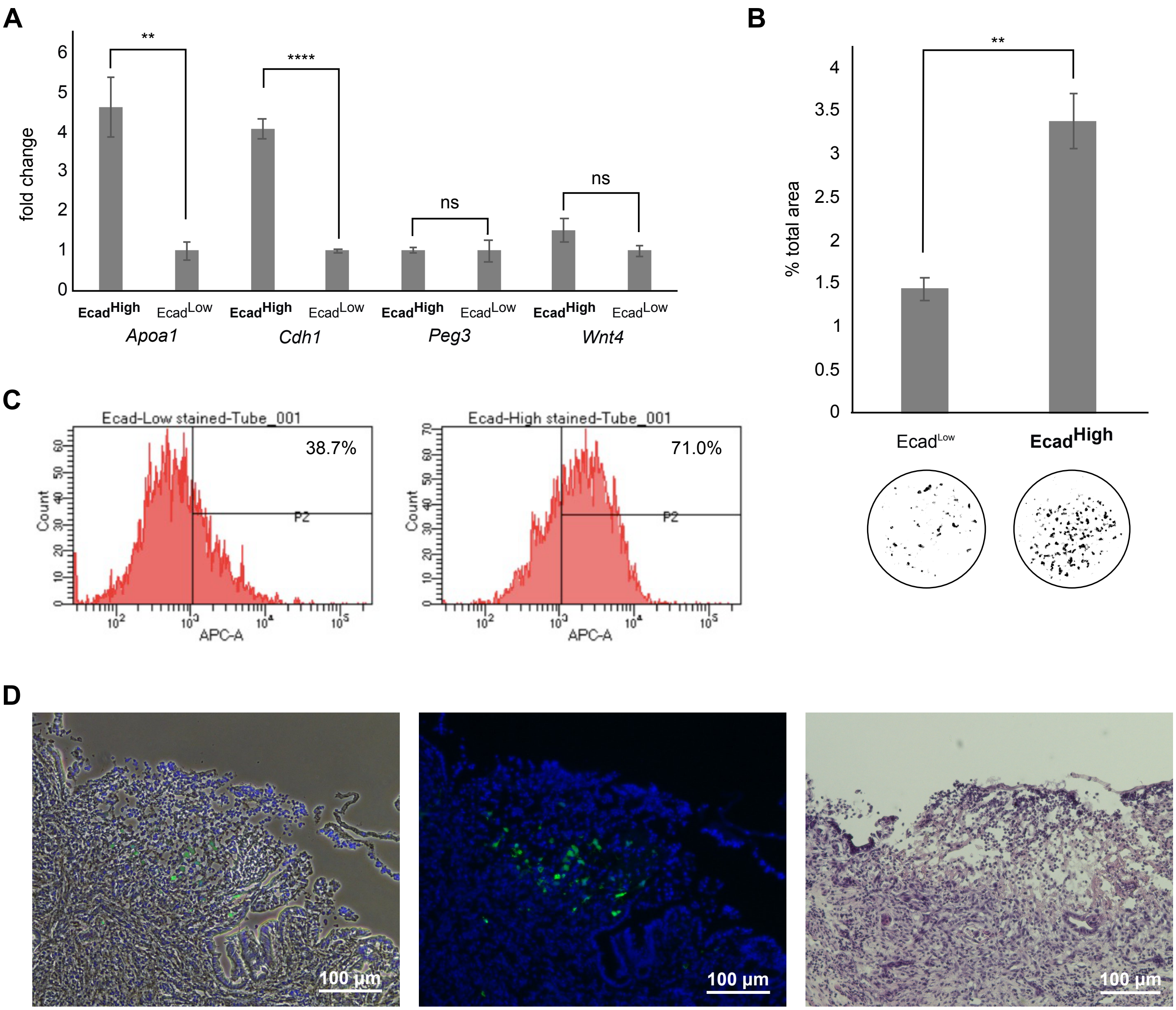

A
fold change
0
1
2
3
4
5
6
**
****
ns
ns
EcadHigh
EcadLow
Apoa1
Cdh1
Peg3
Wnt4
B
% total area
0
0.5
1
1.5
2
2.5
3
3.5
4
**
EcadLow
EcadHigh
C
Ecad-Low stained-Tube_001
38.7%
Ecad-High stained-Tube_001
71.0%
Count
APC-A
P2
D
100 µm
100 µm
100 µm

Fig. S4 Successfully reprogrammed, $Apoa1^{High}Ecad^{High}$ iEPs can be enriched by sorting by E-cadherin expression into $Ecad^{High}$ and $Ecad^{Low}$ iEPs. **A,** qRT-PCR of $Ecad^{High}$ and $Ecad^{Low}$ iEPs show overexpression of iEP markers *Apoa1* and *Cdh1* in $Ecad^{High}$ iEPs. **B,** Quantification of colony formation assay of $Ecad^{High}$ and $Ecad^{Low}$ iEPs shows that $Ecad^{High}$ iEPs have a statistically significantly higher colony area as a proportion of total area. Bottom, threshold images of colonies. $Ecad^{High}$ iEPs have a 1.44-fold higher colony count compared to $Ecad^{Low}$ iEPs. **C,** Sorted $Ecad^{High}$ and $Ecad^{Low}$ iEPs were plated and cultured for one week. Flow cytometry analysis of cultured $Ecad^{High}$ and $Ecad^{Low}$ iEPs confirms that $Ecad^{High}$ iEPs retain their $Ecad^{High}$ phenotype. **, $p < 0.01$. ****, $p < 0.0001$. ns, non-significant. **D,** $Ecad^{High}$ and $Ecad^{Low}$ iEPs were labeled with CellTagA and CellTagB, respectively, and pooled in equal proportions for transplantation into the mouse colon. Left and Middle, bright field and fluorescent images of DAPI stained colon section showing aggregated GFP^{+} iEPs near the surface of the damaged epithelium. Right, H&E staining of an adjacent section showing epithelial injury and inflammation with numerous lymphocytic infiltrates. Scale bars, 100 μm.

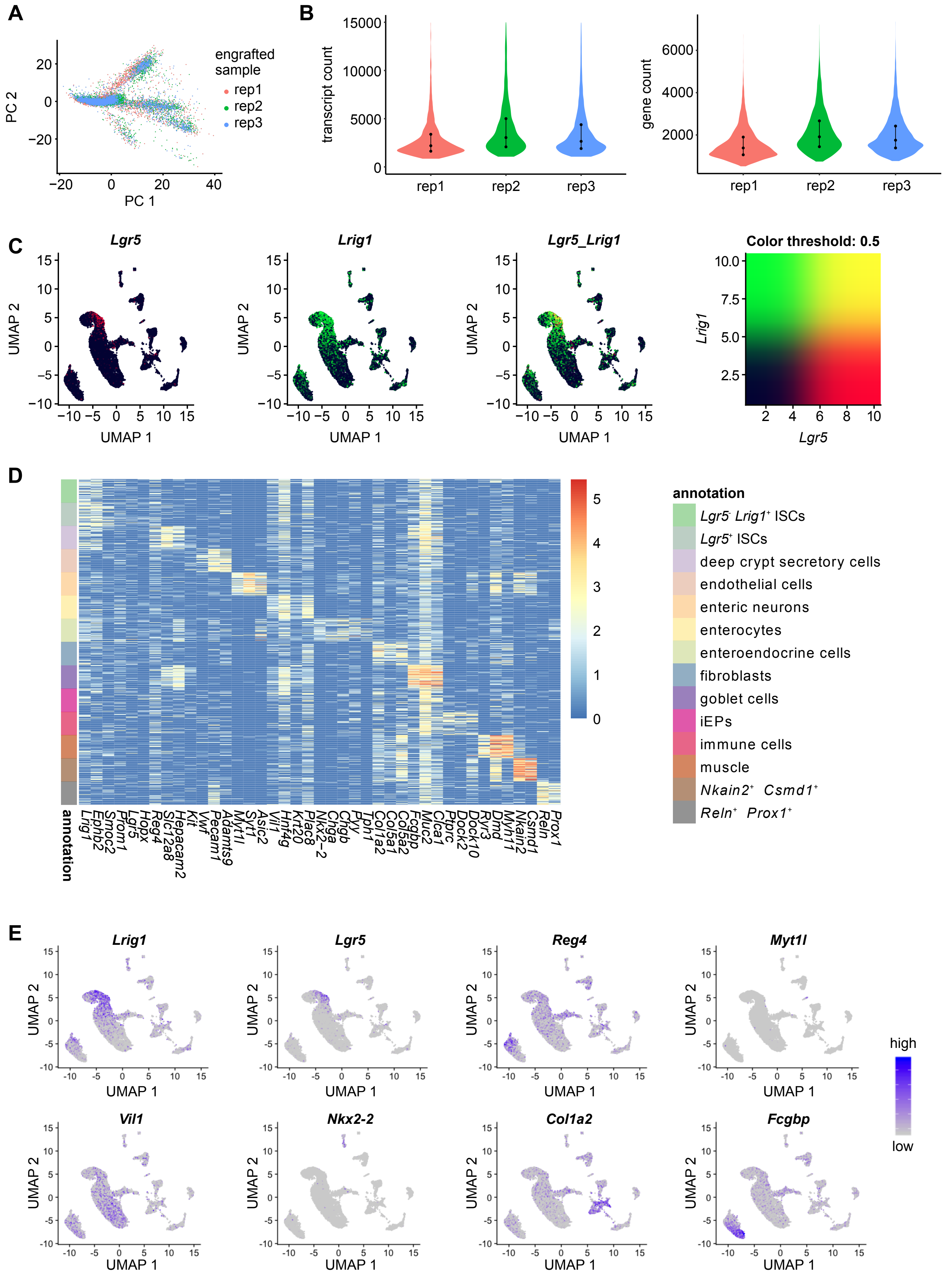
A
B
C
D
E
PC 2
PC 1
engrafted sample
rep1
rep2
rep3
transcript count
gene count
Lgr5
Lrig1
Lgr5_Lrig1
Color threshold: 0.5
UMAP 1
UMAP 2
annotation
Lgr5- Lrig1+ ISCs
Lgr5+ ISCs
deep crypt secretory cells
endothelial cells
enteric neurons
enterocytes
enteroendocrine cells
fibroblasts
goblet cells
iEPs
immune cells
muscle
Nkain2+ Csmd1+
Reln+ Prox1+
Lrig1
Ephb2
Smoc2
Prom1
Lgr5
Hopx
Reg4
Slc12a8
Hepacam2
Kit
Vwf
Pecam1
Adamts9
Myt1l
Syt1
Asic2
Vil1
Hnf4g
Krt20
Plac8
Nkx2−2
Chga
Chgb
Fyb
Ptprc
Tph1
Col1a2
Col5a1
Col5a2
Fcgbp
Muc2
Clca1
Ptprc
Dock2
Dock10
Ryr3
Dmd
Myh11
Nkain2
Csmd1
Reln
Prox1
Reg4
Myt1l
Vil1
Nkx2-2
Col1a2
Fcgbp
high
low

Fig. S5 Single-nucleus RNA-seq of iEP-engrafted colon tissues reveals intestinal cell types. **A,** Visualization of three biological replicates of engrafted colon (rep1, rep2, rep3) integrated into a single dataset, projected onto PC 1 and P C2. **B,** Engrafted samples share similar levels of total numbers of transcript and gene detected per cell. **C,** 'Blended' feature plots of *Lgr5* and *Lrig1* expression, showing a pattern of *Lrig1* expression partially overlapping with areas with high *Lgr5* expression. **D,** Heatmap of intestinal epithelial and non-epithelial marker expression in annotated cell types (50 cells randomly sampled from each cell type). **E,** Additional feature plots of intestinal marker expression.

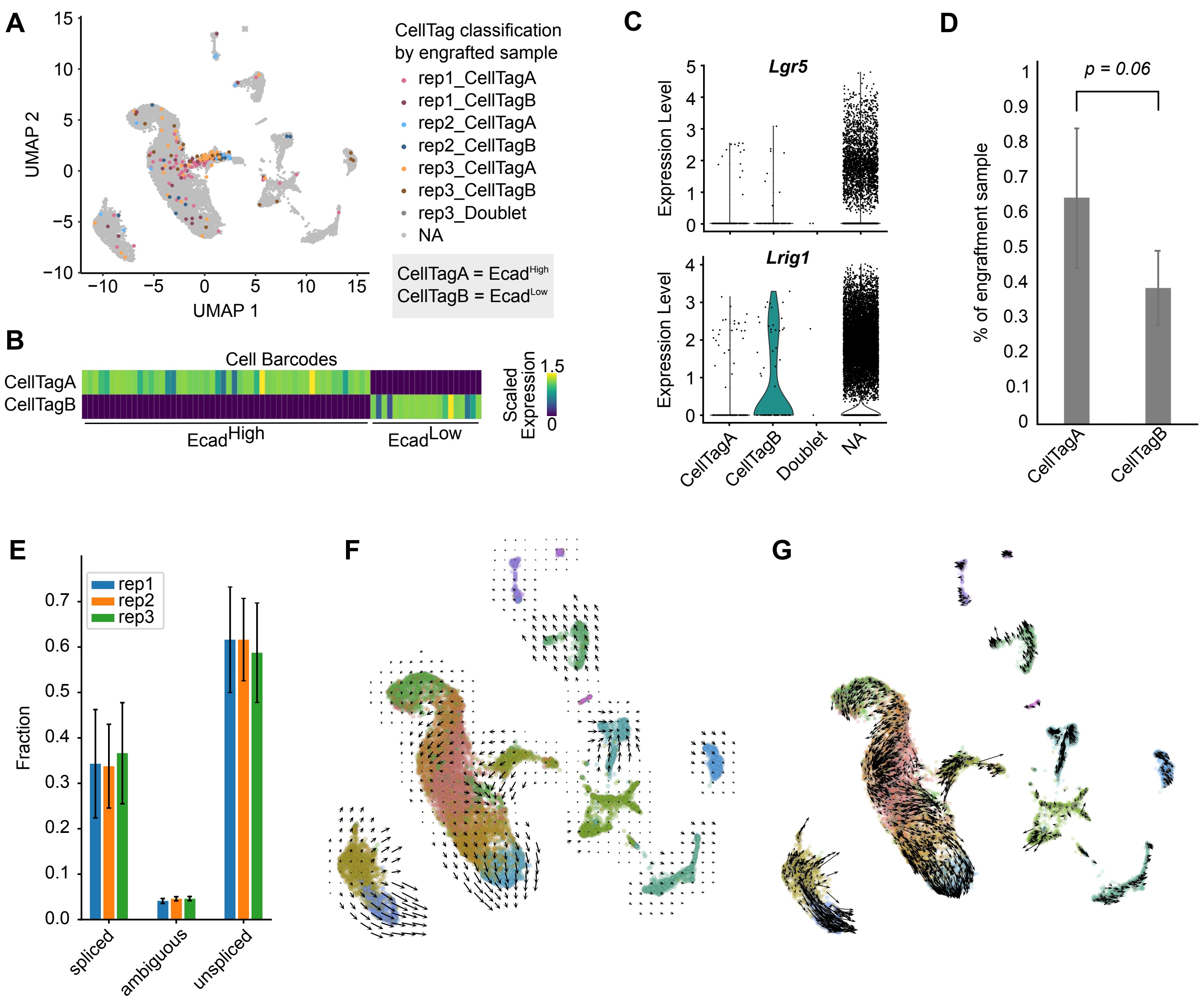
A
CellTag classification by engrafted sample
rep1_CellTagA
rep1_CellTagB
rep2_CellTagA
rep2_CellTagB
rep3_CellTagA
rep3_CellTagB
rep3_Doublet
NA
CellTagA = Ecad High
CellTagB = Ecad Low
UMAP 1
UMAP 2
B
Cell Barcodes
CellTagA
CellTagB
Ecad High
Ecad Low
Scaled Expression
C
Lgr5
Lrig1
Expression Level
CellTagA
CellTagB
Doublet
NA
D
p = 0.06
% of engraftment sample
CellTagA
CellTagB
E
rep1
rep2
rep3
Fraction
spliced
ambiguous
unspliced
F
G

Fig. S6 Visualization of additional CellTag and RNA velocity analysis in iEP-engrafted colon. **A,** CellTag classification shows agreement between three biological replicates of engrafted colon (rep1, rep2, rep3). **B,** Heatmap of scaled expression of CellTagA and CellTagB from rep1 shows distinct patterns of expression. **C,** 0.687% ± 0.214% of each post-engraftment sample were derived from $Ecad^{High}$/CellTagA cells, whereas 0.413% ± 0.113% were derived from $Ecad^{Low}$/CellTagB cells. One-sided student's t-test, p = 0.06. **D,** *Lgr5* and *Lrig1* expression is detected in a subset of CellTagged cells. **E,** Post-engraftment samples share similar transcriptional kinetics with indistinguishable proportions of spliced and unspliced transcripts. **F,** Full vector field of RNA velocity results. **G,** Full velocity vectors of RNA velocity results.
